# Vitamin D Status and Pregnancy Complications: Serum 1,25-di-hydroxyl-Vitamin D and its Ratio to 25-hydroxy-Vitamin D are Superior Biomarkers than 25-hydroxy-Vitamin D

**DOI:** 10.7150/ijms.47807

**Published:** 2020-10-18

**Authors:** Ibrahim A. Albahlol, Abdulrahman H. Almaeen, Abdulrahman A. Alduraywish, Umar F. Dar, Tarek H. El-Metwally

**Affiliations:** 1 Department of Obstetrics and Gynecology, College of Medicine, Jouf University, Sakaka, Saudi Arabia. iaelbahloul@ju.edu.sa; 2 Department of Obstetrics and Gynecology, Mansoura University, Mansoura, Egypt.; 3 Department of Pathology, College of Medicine, Jouf University, Sakaka, Saudi Arabia. ahalmaeen@ju.edu.sa; 4 Department of Internal Medicine, College of Medicine, Jouf University, Sakaka, Saudi Arabia. dr-aaad@ju.edu.sa; 5 Department of Family and Community Medicine, College of Medicine, Jouf University, Sakaka, Saudi Arabia. ufdar@ju.edu.sa; 6 Department of Pathology, Biochemistry Division, College of Medicine, Jouf University, Sakaka, Saudi Arabia. thelmetwally@ju.edu.sa.; 7 Department of Medical Biochemistry, Faculty of Medicine, Assiut University, Assiut, Egypt.

**Keywords:** Serum vitamin D biomarkers, pregnancy outcomes, pregnancy complications, 25-hydroxyvitamin D, 1, 25-dihydroxyvitamin D

## Abstract

Vitamin D (VitD) deficiency during pregnancy has been associated with adverse neonatal outcomes and increased risk of late pregnancy complications. We planned to correlate serum VitD biomarkers; 25-hydroxyvitamin D (25-OH-VitD) and 1,25-dihydroxyvitamin D (1,25-diOH-VitD) levels; and their ratio with the frequency of feto-maternal pregnancy complications. A prospective cross-sectional case-control study was conducted at Aljouf Maternity and Children Hospital, Sakaka, Saudi Arabia, during the period of September 1, 2017 to September 30, 2019. 322 pregnant women were stratified into 2 groups: controls (110 cases) and complicated group (212 cases). The later comprised severe preeclamptic toxemia associated with intrauterine growth restriction (58 cases), gestational diabetes mellitus (GDM; 82 cases), abortion (26 cases), undisturbed ectopic pregnancy (16 cases), premature rupture of membranes (PROM; 14 cases), and, inevitable preterm labour (16 cases). After clinical assessment, peripheral blood samples were collected. Serum biomarkers were measured using specific immunoassays. The direct 1,25-diOH-VitD/25-OH-VitD ratio was calculated. Serum 25-OH-VitD indicated widely spreading VitD deficiency among participants with significantly higher levels in controls vs. GDM subgroup only. 1,25-diOH-VitD levels and the ratio were markedly reduced in the six complicated subgroups vs. controls, with non-significant differences amongst the complicated subgroups. ROC analysis showed very high sensitivity and specificity, to differentiate patients from controls, only for 1,25-diOH-VitD (AUC = 0.965; 0.947 - 0.983, p <0.001) followed by the ratio but not 25-OH-VitD. In conclusions, 25-OH-VitD did not show significant changes except for GDM. 1,25-diOH-VitD levels and the ratio showed strong associations with pregnancy complications. Serum 1,25-di-OH-VitD and its ratio to 25-OH-VitD are more reliable and physiologically relevant biomarkers for VitD status in pregnancy.

## Introduction

Vitamin D (VitD) deficiency became a global epidemic, particularly for women of reproductive age. Nuclear receptor-mediated, the dihydoxy active form of VitD, 1,25-diOH-VitD, controls cellular proliferation, differentiation and apoptosis through targeting ≥1000 genes. Nevertheless, pathogenetic role of hypovitaminosis D in a number of comorbidities is still hotly debated 1,2. With increased requirements of the conditional VitD, pregnancy is a high-risk factor for VitD deficiency since the fetus gains its VitD requirements from mother 3. Despite abundant sunlight, published reports confirmed a wide spread of hypovitaminosis D among Saudi citizens 4. More than half of UK population is VitD deficient, i.e., having serum 25-hydroxy-VitD (25-OH-VitD) <20 ng/mL - as defined by the Endocrine Society 5. To maintain healthy musculoskeletal system, the Scientific Advisory Committee on Nutrition Department recommended a serum 25-OH-VitD cut-off value of 10 ng/mL for all persons 6.

Linked to hypovitaminosis D, adverse fetal outcomes included abortion, intrauterine growth restriction, fetal death and congenital malformations. Maternal adverse effects involved preeclampsia, gestational diabetes mellitus (GDM) and increased risk of preterm labor 7-9. Although the causative role of VitD deficiency in different pregnancy complications is still an open question - due to the insufficient reports locally and globally, VitD supplementation may reduce GDM, preeclampsia, preterm labor, and subnormal neonatal anthropometric measures 10. Meta-analyses of observational studies correlate hypovitaminosis D with pregnancy complications without asserting causation 11. The longitudinal pattern of changes in 25-OH-VitD concentrations and its relationship with 1,25-diOH-VitD throughout pregnancy remains largely unclear. Time-dependent decreases, increases or no change in 25-OH-VitD levels were reported, while 1,25-diOH-VitD levels mostly increase at term compared to non-pregnant women. Additionally, serum VitD cutoffs that are defined for the general adult population are inappropriately used for pregnancy 3.

We planned to assess the relationship between hypovitaminosis D and feto-maternal pregnancy complications and morbidities, using the more patho-physiologically relevant biomarkers. We measured serum 25-OH-VitD, as the established VitD biomarker, and, 1,25-diOH-VitD as a functional VitD biomarker, and their ratio, and, correlated them with the existing pregnancy feto-maternal status.

## Materials and Methods

### Settings and participants

This prospective cross-sectional case control study was carried out at Aljouf Maternity and Children Hospital, Sakaka, Saudi Arabia, during the period between September 1, 2017 and September 30, 2019. It was approved by the local bioethical committees of Jouf University and Ministry of Health (#6-16-4/40). Written informed consents were obtained. The exclusion criteria were uncertain diagnosis, obesity, anemia, endocrinal disorders (hypo-/hyper-parathyroidism and thyroidism, and diabetes other than GDM), immobilization for any reason, on anti-convulsion drug, acute infections, malabsorption syndromes, comorbid chronic medical disorder (renal or liver impairment, inflammatory and immunological disorders), multiple pregnancy, antepartum hemorrhage and superimposed preeclampsia.

### Examination and investigations

Among 450 pregnant women attending the hospital, 322 voluntary participants fulfilling the inclusion criteria were recruited. The 322 participants were classified into 2 groups; control group of 110 women who had normal feto-maternal pregnancy, and, complicated pregnancy group comprising 212 participants. The latter group was further subdivided into: severe preeclamptic toxemia (PET) associated with intrauterine growth restriction (IUGR; 58 cases), GDM (82 cases), abortion (26 cases), undisturbed ectopic pregnancy (16 cases), premature rupture of membranes (PROMs; 14 cases), and, inevitable preterm labor (16 cases).

After history taking (for age, gravidity, parity, duration of pregnancy in weeks and significant medical history) and general examination (for vital signs and weight and height to calculate body mass index; BMI), the head, face, neck, chest, heart, back, lower limbs and abdomen were systematically examined. The pregnancy was assessed ultra-sonographically for viability and fetal biometry to determine pregnancy duration and the expected fetal weight, condition of amniotic fluid and for diagnosing fetal anomalies, if present. Women with routine laboratory findings that contradict the inclusion criteria were excluded.

Serum was recovered from peripheral blood samples by centrifugation and was aliquotted and stored till used at -80 ^o^C. Specific quantitative ELISA immunoassay kits (Sunlong Biotech Co. Ltd., Zhejiang, China) were used to measure total 25-OH-VitD (in ng/mL; cat# SL2762Hu) and 1,25-DiOH-VitD (in pg/mL; cat# SL2845Hu). The direct 1,25-DiOH-VitD/25-OH-VitD ratio was calculated for each patient. Participants were stratified using the clinically relevant 25-OH-VitD cutoff levels of high/toxic as ≥50/>80 ng/mL, normal as ≥30-50 ng/mL, insufficient as ≥20 - <30 ng/mL, deficient as ≥10 - <20 ng/mL and severely deficient as <10 ng/mL 5, 12-14. Unfortunately, all of the cutoff recommendations do not refer to pregnancy and its complication as distinct clinical entities.

### Statistical analysis

Data was analyzed using SPSS (Statistical Package for Social Sciences, Version 23.0. Armonk, NY: IBM Corp). We expressed descriptive qualitative data as frequency and percentage, and, quantitative data as range and mean ± standard deviation (SD). Normal distribution was checked by Kolmogorov-Sminov test. One-way ANOVA with Tukey's post-test was employed for multiple comparisons. Correlation among variables was assessed by Spearman correlation coefficient test. Receiver Operating Characteristic (ROC) curve analysis was used to determine the area under curve (AUC) for 25-OH-VitD, 1,25-DiOH-VitD and their ratio to check their sensitivity and specificity to differentiate the cases from controls. P value of <0.05 at a confidence level of 95% was considered significant.

## Results

### Demographics, anthropometrics, clinical characteristics and vitamin D biomarkers (Table 1)

Age was nonsignificantly different among all groups except for preeclamptics vs. each of controls (p = 0.011) and PROMs (p = 0.014), and, PROMs vs. GDM (p = 0.035). BMI showed nonsignificant difference among the investigated groups except comparing GDM vs. each of controls (p = 0.002), abortion (p = 0.02), ectopic pregnancy (p <0.001) and PROMs (p <0.001); and preeclamptics vs. each of ectopic pregnancy (p <0.028) and PROM (p = 0.045). Gravidity showed nonsignificant difference among the investigated groups except comparing GDM vs. each of controls (p <0.001), preeclamptics (p <0.001), abortion (p <0.036), ectopic pregnancy (p = 0.002) and PROMs (p = 0.002). Parity showed nonsignificant difference among the investigated groups except comparing GDM vs. each of preeclamptics (p <0.001), abortion (p = 0.035), ectopic pregnancy (p = 0.002) and PROMs (p <0.035). Duration of pregnancy showed very strong significant difference among the investigated groups with a few non-significant exceptions; it was significantly different comparing controls vs. all complication subgroups (p <0.01 - 0.001) except GDM, comparing preeclamptics vs. all other subgroups (p <0.05 - <0.001) except preterm labour, comparing GDM vs. the others (p <0.001), comparing abortion vs. the others (p <0.001) except ectopic pregnancy, and, comparing ectopic pregnancy vs. the others (p <0.001) except PROMs.

Serum 25-OH-VitD levels showed nonsignificant difference among the investigated groups except comparing GDM vs. each of controls (p <0.001) and ectopic pregnancy (p = 0.01). Serum 1,25-diOH-VitD levels showed nonsignificant difference among the investigated groups of patients. However, controls had markedly higher levels vs. each of the six complicated groups (p <0.001). Similarly, the direct 1,25-diOH-VitD/25-OH-VitD ratio showed very marked significant reduction comparing controls vs. each of the six patients' groups (p <0.001), albeit, with nonsignificant difference among the complicated groups.

### Stratification of our participants on bases of the clinical 25-OH-VitD cutoff values (Table 2)

Using serum 25-OH-VitD as the biomarker for VitD status, 43.636% of controls were deficient (≥10 ng/mL), 38.181% were insufficient (≥20 ng/mL), and, equal proportions (9.09%) were either normal (≥30 ng/mL) or having toxic levels (>80 ng/mL). 82.759% of preeclamptic cases were insufficient, 13.793% were deficient and 3.448% had normal levels. 82.927% of GDM cases were deficient, 12.195% were insufficient and 4.878 had normal levels. 84.615% of abortion cases deficient, and equal proportions (7.692%) had either insufficient or toxic levels. 50.0% of ectopic pregnancy cases were deficient, 37.5% were insufficient and the rest (12.5%/2) had toxic levels. 71.429%/ of PROMs cases were deficient and 28.571% were insufficient. 75% of preterm labor cases were insufficient and 25.0% were deficient. Fortunately, severe deficiency (<10 ng/mL) was not observed in our cases. Therefore, VitD insufficiency/deficiency is widely spreading among the study participants (292 out of 322, i.e., 90.683%). Controls presented a better picture only considering insufficiency/deficiency proportion, and, % of normal/super-normal levels.

### ROC curve analysis

**Figure 1:**
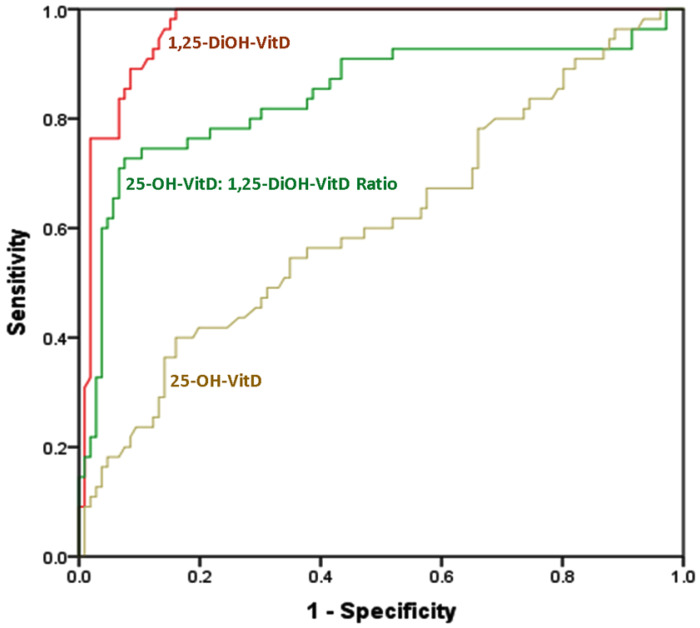
The ROC curve for sensitivity and specificity of each of 1,25-DiOH-VitD levels, 1,25-DiOH-VitD: 25-OH-VitD ratio, and, 25-OH-VitD levels for differentiation between cases (n = 212) and controls (n = 110) among Saudi women with and without pregnancy complications. Diagonal segments were produced by ties.

ROC curve analysis showed that 1,25-DiOH-VitD level is the most sensitive and most specific biomarker for differentiating between cases and controls; with an AUC of 0.965 (0.947 - 0.983, p <0.001). It is followed by 1,25-DiOH-VitD25-OH-VitD ratio; with AUC of 0.843 (0.791 - 0.676, p <0.001). 25-OH-VitD levels fell near the diagonal line with no effect, i.e., showing least sensitivity and least specificity (AUC = 0.61; 0.543 - 0.676, and, p = 0.001) (Figure 1 and Table 3).

### Results of the correlation analysis (presented as r/p values)

For controls, age showed positive correlations vs. each of gravidity (0.514/<0.001), parity (0.468/<0.001) and BMI (0.837/<0.001), and negative relationship with the ratio (-0.155/<0.05). Gravidity showed positive relationship vs. parity (0.958/<0.001) and BMI (0.480/<0.001). Parity correlated positively with BMI (0.448/<0.001). BMI correlated negatively vs. the ratio (-0.184/= 0.027). Pregnancy duration correlated negatively with 1,25-DiOH-VitD (-0.241/<0.006) and ratio (-0.192/= 0.022). 25-OH-VitD correlated positively vs. 1,25-DiOH-VitD (0.157/<0.05) and negatively vs. the ratio (-0.647/<0.001). 1,25-DiOH-VitD correlated positively with the ratio (0.562/<0.001).

For preeclamptic cases, age had positive correlation vs. gravidity (0.682/<0.001), parity (0.608/<0.001) and BMI (0.690/<0.001), and, negative correlation vs. 1,25-DiOH-VitD (-0.267/= 0.021). Gravidity related had positively with parity (0.962/<0.001) and BMI (0.497/<0.001), but, negatively vs. 1,25-DiOG-VitD (-0.282/= 0.016). Parity correlated positively vs. BMI (0.424/<0.001), and, negatively with 1,25-DiOH-VitD (-0.218/<0.05). BMI correlated positively vs. duration of pregnancy (0.333/= 0.005) and negatively vs. 1,25-DiOH-VitD (-0.275/= 0.017). 25-OH-VitD correlated negatively with the ratio (-0.490/<0.001). 1,25-DiOH-VitD correlated positively vs. the ratio (0.853/<0.001).

For GDM cases, age correlated positively vs. each of gravidity (0.680/<0.001), parity (0.732/<0.001), BMI (0.657/<0.001) and 25-OH-VitD (0.239/= 0.015), but, negatively vs. each of 1,25-DiOH-VitD (-0.179/<0.05) and the ratio (-0.320/<0.002). Gravidity correlated positively with parity (0.865/<0.001), BMI (0.671/<0.001) and 25-OH-VitD (0.216/<0.026), and, negatively vs. duration of pregnancy (-0.294/<0.004) and the ratio (-0.183/<0.05). Parity correlated positively vs. BMI (0.725/<0.001) and 25-OH-VitD (0.187/0.047), and, negatively vs. duration of pregnancy (-0.179/<0.05). BMI correlated positively vs. 25-OH-VitD (0.412/<0.001) and negatively vs. the ratio (-0.225/<0.021). 25-OH-VitD correlated negatively with the ratio (-0.757/<0.001). 1,25-DiOH-VitD had positive correlation vs. the ratio (0.592/<0.001).

For abortion cases, age correlated positively with gravidity (0.406/<0.02), parity (0.535/= 0.0024), BMI (0.757/<0.001), and pregnancy duration (0.337/= 0.046), and, negatively vs. 1,25-DiOH-VitD (-0.669/<0.001) and the ratio (-0.380/<0.028). Gravidity correlated positively with parity (0.815/<0.001) and duration of pregnancy (0.565/= 0.0013), and, negatively vs. each of 1,25-DiOH-VitD (-0.333/= 0.048) and the ratio (-0.381/ = 0.027). Parity related positively with duration of pregnancy (0.358/= 0.036). BMI associated negatively with 1,25-DiOH-VitD (-0.385/= 0.026). Pregnancy duration correlated negatively vs. 1.25-DiOH-VitD (-0.371/<0.031) and the ratio (-0.490/<0.006). 25-OH-VitD correlated negatively vs. the ratio (-0.773/<0.001). 1,25-DiOH-VitD correlated positively vs. the ratio (0.665/<0.001).

For the ectopic pregnancy cases, age correlated positively vs. gravidity (0.884/<0.001), parity (0.927/<0.001), and BMI (0.922/<0.001), but, negatively vs. 1,25-DiOH-VitD (-0.639/<0.005) and the ratio (-0.527/<0.02). Gravidity correlated positively vs. parity (0.906/<0.001) and BMI (0.854/<0.001), and, negatively vs. 1,25-DiOH-VitD (-0.565/<0.013). Parity correlated positively with BMI (0.933/<0.001), and, negatively vs. 1,25-DiOH-VitD (-0.624/<0.006) and the ratio (-0.437/<0.05). BMI correlated positively vs. duration of pregnancy (0.433/<0.05), and, negatively vs. 1,25-DiOH-VitD (-0.443/= 0.044). Pregnancy duration related negatively vs. 1,25-DiOH-VitD (-0.504/<0.027). 25-OH-VitD correlated negatively vs. the ratio (-0.690/= 0.002). 1,25-DiOH-VitD correlated positively with the ratio (0.647/= 0.004).

Among PROMs cases, age correlated positively vs. gravidity (0.578/<0.018), parity (0.578/<0.018), BMI (0.727/= 0.002), 1,25-DiOH-VitD (0.500/<0.038) and the ratio (0.474/<0.05). Gravidity correlated positively vs. parity (1.0/<0.001), BMI (0.517/= 0.03) and duration of pregnancy (0.459/<0.05), but, negatively vs. 25-OH-VitD (-0.543/<0.026). Parity associated positively vs. BMI (0.517/= 0.03) and pregnancy duration (0.467/<0.05), but, negatively vs. 25-OH-VitD (-0.543/<0.026). BMI positively correlated with 1,25-DiOH-VitD (0.800/<0.001) and the ratio (0.655/= 0.007). 25-OH-VitD correlated positively with 1,25-DiOH-VitD (0.468/<0.048). 1,25-DiOH-VitD correlated positively vs. the ratio (0.786/<0.001).

Within the preterm labor cases, age correlated positively vs. gravidity (0.898/<0.001), parity (0.908/<0.001), BMI (0.952/<0.001), and the ratio (0.452/<0.042), but, negatively vs. 25-OH-VitD (-0.439/<0.05). Gravidity correlated positively vs. parity (0.933/<0.001), BMI (0.802/<0.001) and the ratio (0.503/= 0.025). Parity correlated positively vs. BMI (0.835/<0.001) and the ratio (0.454/= 0.04), but, negatively with 25-OH-VitD (-0.454/= 0.04). BMI associated negatively with 25-OH-VitD (-0.500/<0.027), but, positively vs. the ratio (0.548/= 0.016). 1,25-DiOH-VitD correlated positively vs. the ratio (0.786/<0.001).

## Discussion

Age was insignificantly different among our complicated groups. It had significant difference only when controls are compared to preeclamptics. Preeclampsia usually attacks extremely aged mothers; mainly primigravida. Gravidity showed a significant difference between the controls and GDM groups. Repeated pregnancy is a definite risk factor for GDM development. BMI was significantly different only comparing GDM and controls. GDM usually associates with higher BMI. As the gestational age is unique for the occurring complication (e.g., preeclampsia develops after 20 weeks of gestation, abortion frequently happens in the 1^st^ trimester of pregnancy, PROMs occurs after 37^th^ weeks and before onset of labor and GDM is common at beginning of the last trimester), it showed significant differences comparing all groups 15-19.

Considering the association between VitD deficiency and risk of feto-maternal pregnancy complications, the bending and intriguing questions are: How to achieve and specify the optimal pregnancy VitD level? What optimum biomarker and cutoffs to measure?, and, how to determine the real benefits of VitD supplementation in pregnancy? 20. Even implementing a personal physiological response-dependent VitD signature turned confusing due to varying physiological responses at same supplementation dose and increment plasma 25-OH-VitD levels 21. Reports related VitD level to clinical morbidities using serum 25-OH-VitD as a VitD biomarker - based on its higher stability and longer biological half-life. Such validity is substantiated by the significant correlation of serum 25-OH-VitD with non-calcemic clinical outcomes and its existence at high cellular levels. Both assumptions are not unquestionably correct 22, 23. Indeed, the present study and a long list of previous studies, including ours, had shown that serum 25-OH-VitD does not differentiate apparently healthy controls from patients with different comorbidities including pregnancy, diabetes and infection 4, 8, 24-31. This could be partially due to our and others observation of widely spreading hypovitaminosis D among population 4, 11. 25-OH-VitD is produced from several sources and leak to serum without direct reflection of cellular contents 32, 33.

Serum level of 1,25-diOH-VitD was considered a less accurate predictor of intracellular VitD concentration. This came from the notion that 1,25-diOH-VitD intracellular level exceeds its serum level by several folds along with extra-renal sources for it 22. Indeed, cells have concentrating modalities for lipophilic ligands. Moreover, serum 1,25-diOH-VitD is already VDR receptor saturating. Therefore, the intracellular receptor supersaturating concentration is not required, albeit, for potential VDR-independent actions 34. We resourced 1,25-diOH-VitD since several studies reported its superiority over 25-OH-VitD as an independent clinical correlate 31, 35-37. 25-OH-VitD is the local precursor and increases in its circulating levels parallel consequent increases in 1,25-diOH-VitD however, their relationship is not always straightforward 30, 38. For instance, adequate circulating 1,25-diOH-VitD levels are necessary for sufficient 25-OH-VitD availability in VitD target cells, e.g., monocytes 39. Moreover, pregnant Brazilian women insufficient in VitD at baseline had higher increases in 1,25-diOH-VitD concentrations, over pregnancy time, compared to women with sufficient VitD levels 3. VitD supplementation do not increase rate of sufficiency in all occasions 40. However, 1,25-diOH-VitD levels had no seasonal variation like that of 25-OH-VitD in healthy Canadians 41. Moreover, a study found a strong correlation between circulating 1,25-diOH-VitD and 25-OH-VitD levels throughout pregnancy and that 1,25-diOH-VitD levels at 12 weeks' gestation are approximately triple that of normal nonpregnant female 42.

Our controls were overwhelmingly VitD insufficient which is in accord with another Saudi study reporting 3.5% VitD sufficiency rate among pregnant women 43. However, significantly lower 25-OH-VitD level vs. controls was recorded only for our GDM women among other complications. Ironically, those with ectopic pregnancy showed nonsignificant higher, while, other complications had nonsignificantly lower levels than controls. Grouping deficient and insufficient subjects revealed decreasing serum 25-OH-VitD rates as follows: Controls, ectopic pregnancy, abortion, GDM, preeclampsia, preterm labour, and PROMs. Oppositely, 1,25-diOH-VitD levels showed marked reductions in each of 6 complicated groups vs. controls. The complicated women groups were nonsignificantly different. Changes in 1,25-diOH-VitD/25-OH-VitD ratio mirrored 1,25-diOH-VitD pattern. While sensitivity and specificity of both of 1,25-diOH-VitD and the ratio were very high, those of 25-OH-VitD were very low in differentiating between our controls and complicated pregnancy. Pregnancy hypovitaminosis D in developing countries and its associated adverse feto-maternal hazards is a commonplace 10, 44. Similar to our GDM women, studies had correlated hypovitaminosis D higher risk of development of GDM, miscarriage and still birth in the 2^nd^ and 3^rd^ trimester 23, 45-47. Despite the wide spreading 25-OH-VitD deficiency in controls (71.2 vs. 83.3%), women with GDM had a 2.66-fold increased risk of being deficient status 48.

Other studies conducted in Saudi Arabia, USA and China did not find association between adverse pregnancy outcomes and low 25-OH-VitD level except for high prevalence of abortions among other outcomes 8, 24, 26. Surprisingly, the Chinese study found higher prevalence of GDM and preterm delivery among pregnant women with high 25-OH-VitD level despite older maternal age and higher BMI 18. Actually 5% of women with GDM of our study had normal VitD levels. Rodriguez et al reported no association of VitD concentration and GDM, preterm delivery and IUGR 25. Also, hypovitaminosis D in early pregnancy did not associate with adverse pregnancy outcomes 26. GDM had no association with vitamin D deficiency 27, 28.

Similar to the trend of our results, higher risks of development of preeclampsia, GDM, preterm and cesarean delivery and IUGR were related to hypovitaminosis D 49, 50. Normal 25-OH-VitD levels decrease the risk of preeclampsia and IUGR 51. To establish a causal relationship, large VitD supplementation RCTs must guarantee optimum feto-maternal outcomes 52. On the contrary, vitamin D supplementation did not affect the incidence of adverse pregnancy outcomes like preeclampsia, GDM and IUGR 53. Conflictingly, supplementation with VitD in early pregnants diminished GDM incidence in pregnant with hypovitaminosis D 54. Increased 25-OH-VitD levels after VitD supplementation correlate with significant reduction in rate of preterm labour, pre-eclampsia, and GDM in pregnant Indian women 43. Women with persistent 25-OH-VitD deficiency up to 26^th^ weeks of gestation had a 4.46-fold elevated risk for GDM 55. Significant reduction in GDM risk was observed with increasing 25-OH-VitD levels 56.

Among our preeclampsia patients, 97% were VitD deficient/insufficient. Hypovitaminosis D is a risk factor for severe preeclampsia and season-dependent variation in incidence of preeclampsia seems to correlate with VitD level 57. Low 25-OH-VitD levels and placental VitD receptor expression negatively correlated with preeclampsia 58. 92% of our abortion patients had VitD deficiency/insufficiency. Relationship between low VitD levels and miscarriage stems from VitD regulation of genes concerned with implantation, trophoblastic invasion, angiogenesis, immunomodulation, suppression of inflammation and protection from infection 31, 59, 60. 25-OH-VitD levels were lower in non-gravid women with history of pregnancy loss than normal non-gravid and pregnant women 61. Reduced VitD levels among pregnant women were correlated with increased production of proinflammatory cytokines 59. Women with PROMs and preterm labor were 100% VitD deficient/insufficient in our study. Mothers with preterm labor are three times more likely to have insufficiency when compared to full-term mothers 62. Hypovitaminosis D is associated with high prevalence of infections, particularly Gardnerella vaginalis; commonly encountered in cases of PROMs and preterm labor 63-65. VitD deficiency increased susceptibility to microbial infection due to macrophage dysfunction with defect in toll-like mediated action of the antibacterial peptide cathelicidin 66.

BMI inversely correlates with 25-OH-VitD levels at med-gestation and postpartum 45. Our controls had negative correlations contrasting 1,25-diOH-VitD and the ratio vs. pregnancy duration, BMI and 25-OH-VitD, while 25-OH-VitD had no such correlations. Among our preeclamptics, age, gravidity and BMI correlated negatively with 1,25-diOH-VitD. Our GDM group presented negative correlation between age vs. 1,25-diOH-VitD and the ratio, while gravidity and BMI had negative correlation with the ratio. Our abortion cases had negative correlation contrasting age, gravidity and BMI vs. 1,25-diOH-VitD and the ratio. 1,25-diOH-VitD negative correlation vs. age, gravidity, parity and BMI among our ectopics. In our PROMs cases, age and BMI had positive correlation vs. 1,25-diOH-VitD and the ratio, while, gravidity and parity correlated negatively vs. 25-OH-VitD. Our preterm labor cases showed positive correlation between age, gravidity, parity and BMI vs. the ratio but a negative correlation between age, parity and BMI vs. 25-OH-VitD. Reportedly, gestational age correlates positively with 25-OH-VitD levels and had stronger association with 1,25-diOH-VitD 3. Disagreeable, serum 25-OH-VitD level neither increased the risk of preterm birth nor correlated with gestational age or BMI 67. A big VitD supplementation study conducted in Bangladesh, with a widespread VitD deficiency and fetal-infant growth restriction, showed that supplementation from mid-pregnancy to delivery or 6 months postpartum had no significant effect on pregnancy feto-maternal clinical outcomes, despite normalizing 25-OH-VitD levels 68.

Our study is one of the few studies which evaluated the association of variations in 1,25-diOH-VitD and its ratio to 25-OH-VitD with pregnancy complications. Physiologically, 1,25-diOH-Vit-D levels increase with gestational age to reach a maximum in 3^rd^ trimester without increases in 25-OH-VitD levels 30. In our study, while 25-OH-VitD correlated negatively only with GDM, 1,25-diOH-VitD and its ratio to 25-OH-Vit-D correlated negatively with all of pregnancy complications investigated. This may help clearing the discrepancy among the previous studies utilizing 25-OH-Vit-D as the sole VitD biomarker for adverse pregnancy complications. We claimed that addition of 1,25-diOH-VitD and the ratio in evaluating implication in pregnancy complications are clinically meaningful than 25-OH-VitD alone. They both showed higher sensitivity and specificity in differentiating healthy controls vs. complicated pregnancies. However, bigger multi-centric longitudinal and supplementation RCTs are strongly warranted to validate and generalize these assumptions. Several clinical outcomes, including infection, kidney function, diabetes and inflammation, were inversely correlated with circulating 1,25-diOH-VitD levels but not 25-OH-VitD 29, 31, 37.

The norms and biomarkers for VitD levels have to be reevaluated, particularly for pregnancy and its complication, owing to the actual heterogeneity of the world climate and populations. Major potential causes of the widely spreading VitD deficiency we observed include: 1) the traditionally low VitD foods, 2) a scalding local summer sun and ice-cold winter that give very narrow room for sun exposure, 3) low supplement intake, and, 4) absence of a national mandatory VitD food fortification program.

The difficulties faced us carrying out this study included: 1) Absence of a reasonable number of other pregnancy complications such as congenital anomalies, stillbirth, isolated IUGR, 2) we could not correlate findings with comorbid rate of infection and inflammation, length of hospital stays, and long-term outcomes, & 3) Longitudinal analysis was not accessible.

VitD deficiency/insufficiency is widely spreading in the studied population of pregnant women. Levels of 25-OH-VitD were not different among controls and complicated pregnancies except for a significantly lower level in GDM. 25-OH-VitD levels did not correlate with most of the characteristics of participants and pregnancy complications. 1,25-diOH-VitD and its ratio to 25-OH-VitD revealed very highly significant reduction in complicated pregnancy and were highly sensitive and specific in differentiating controls from complicated pregnancies. They also correlate with most of the characteristics of the participating women. 1,25-diOH-VitD and its ratio to 25-OH-VitD are potentially more functionally relevant biomarkers for VitD status particularly during pregnancy.

## Figures and Tables

**Table 1:** Demographic, anthropometric, clinical characteristics, serum 25-hydoxyvitamin D (25-OH-VitD) and 1,25-dihydroxyvitamin D (1,25-DiOH-VitD), and their ratio in Saudi women with and without pregnancy complications**.**

Parameter	Controls n = 110	Preeclampsia n = 58	GDM n = 82	Abortion n = 26	EP n = 16	PROMs n = 14	PTL n = 16
Age, Years	28.51 ± 5.538 (18 - 40)	31.52 ± 5.202 (19 - 42)	31.6 ± 5.7 (22 - 49)	31.2 ± 4.76 (23 - 42)	27.1 ± 6.27 (21 - 40)	26.1 ± 4.82 (19 - 32)	28.5 ± 4.41 (20 - 34)
BMI, kg/m^2^	22.21 ± 1.786 (17.8 - 25.1)	22.6 ± 1.706 (18.8 - 25.2)	23.3 ± 1.93 (18.2 - 25)	21.9 ± 2.06 (17.8 - 25.1)	20.9 ± 2.35 (17.6 - 25.4)	20.9 ± 2.35 (18.4 - 24.2)	22.5 ± 2.02 (18.6 - 24.6)
Gravidity, n	4.055 ± 2.365 (1 - 10)	3.345 ± 1.617 (1 - 6)	5.39 ± 2.07 (1 - 12)	3.92 ± 1.81 (1 - 8)	3.13 ± 1.67 (1 - 6)	3 ± 2.72 (1 - 7)	4 ± 2.42 (1 - 8)
Parity, n	2.691 ± 2.208 (0 - 8)	2.034 ± 1.578 (0 - 5)	3.37 ± 1.55 (0 - 8)	2.08 ± 1.57 (0 - 5)	1.38 ± 1.45 (0 - 4)	1.71 ± 2.27 (0 - 5)	2.13 ± 1.96 (0 - 6)
Pregnancy Duration, Weeks	35.45 ± 5.465 (20 - 41)	31.17 ± 4.849 (22 - 37)	25.1 ± 9.68 (6 - 40)	11.7 ± 4.09 (7 - 18)	6 ± 0.73 (5 - 7)	37.1 ± 2.74 (33 - 39)	33.6 ± 1.93 (29 - 35)
25-OH-VitD, ng/mL	28.46 ± 22.77 (13.48 - 105.6)	23.02 ± 3.674 (15.17 - 35.97)	17.7 ± 4.6 (11 - 35.5)	23.9 ± 19.5 (15.7 - 90.1)	33.6 ± 37 (17.5 - 128)	20 ± 2.59 (17 - 24.7)	20.8 ± 1.17 (18.5 - 22.6)
1,25-DiOH-VitD, pg/mL	111.3 ± 47.54 (49.2 - 281.3)	45.04 ± 9.178 (23.3 - 63.2)	42.4 ± 12.0 (15.9 - 84.0)	45.0 ± 22.4 (32.0 - 119.7)	35.8 ± 7.29 (27.3 - 50.8)	56.6 ± 52.3 (25.8 - 179.3)	34.0 ± 4.29 (27.8 - 41.1)
1,25-DiOH-VitD/25: OH-VitD Ratio	4.956 ± 2.650 (0.956 - 12.9)	1.998 ± 0.533 (1.215 - 3.582)	2.53 ± 0.994 (0.94 - 6.24)	2.34 ± 1.41 (0.485 - 6.71)	1.65 ± 0.72 (0.235 - 2.9)	2.73 ± 2.24 (1.52 - 7.96)	1.63 ± 0.2 (1.34 - 2.0)

Data shown are number of participants per group (n) and mean ± SDM and range. GDM = Gestational diabetes, EP= Ectopic pregnancy, PROMs = Premature rupture of membranes, PTL = Preterm labor. For significance of differences, see the text.

**Table 2:** Serum 25-hydroxyvitamin D levels among Saudi women with and without pregnancy complications.

Group	Toxic (>80)	Normal (≥30 - <50)	Insufficiency (≥20 - <30)	Deficiency (≥10 - <20)	Insf./Defi.
Controls (n = 110)	10 (9.09)	10 (9.09)	42 (38.181)	48 (43.636)	90 (81.818)
Preeclamptics (n = 58)	0 (0)	2 (3.448)	48 (82.759)	8 (13.793)	56 (96.552)
Gestational diabetes (n = 82)	0 (0)	4 (4.878)	10 (12.195)	68 (82.927)	78 (95.122)
Abortion (n = 26)	2 (7.692)	0 (0)	2 (7.692)	22 (84.615)	24 (92.308)
Ectopic Pregnancy (n = 16)	2 (12.5)	0 (0)	6 (37.5)	8 (50.0)	14 (87.5)
PROMs (n = 14)	0 (0)	0 (0)	4 (28.571)	10 (71.429)	14 (100)
Preterm labor (n = 16)	0 (0)	0 (0)	12 (75.0)	4 (25.0)	16 (100)
Total (n = 322)	14 (4.508)	16 (4.969)	124 (38.509)	168 (52.174)	292 (90.683)

Data shown are frequency; n and (%). Insf. = Insufficiency, Defi. = Deficiency. For significance of differences, see the text.

**Table 3:** Area Under the Curve (AUC) for differentiation between cases (n = 212) and controls (n = 110) using each of 1,25-diOH-VitD, 25-OH-VitD: 1,25-diOH-VitD ratio, and, of 25-OH-VitD among Saudi women with and without pregnancy complications.

Test Result Variable(s)	AUC	P value^*^	Range of AUC	
			Lower Bound	Upper Bound
1,25-diOH-VitD	0.965	<0.001	0.947	0.983
1,25-diOH-VitD: 25-OH-VitD ratio	0.843	<0.001	0.791	0.895
25-OH-VitD	0.610	0.001	0.543	0.676

*Null hypothesis: true area = 0.5. Data shown are AUC, p values and AUC range at 95% Confidence Interval.
